# Effectiveness of a Theory-Driven Nutritional Education Program in Improving Calcium Intake among Older Mauritian Adults

**DOI:** 10.1155/2013/750128

**Published:** 2013-12-26

**Authors:** Trishnee Bhurosy, Rajesh Jeewon

**Affiliations:** Department of Health Sciences, Faculty of Science, University of Mauritius, Réduit, Mauritius

## Abstract

*Background*. Low calcium intake, a risk factor of osteoporosis and subsequent fractures, has been previously reported among post-menopausal women in Mauritius. *Objective*. To assess the effectiveness of a theory-based educational intervention in improving the calcium intake, self-efficacy, and knowledge of older Mauritians. *Methodology*. The study was conducted as a pre- and post-test design which was evaluated through a baseline, immediate postintervention, and 2-month follow-up assessments. Participants were adults (*n* = 189) aged ≥40 years old from 2 urban community-based centres. The intervention group (IG) (*n* = 98) participated in 6 weekly interactive lessons based on the health belief model (HBM). The main outcome measures were calcium intake, HB scale scores, knowledge scores, and physical activity level (PAL). Anthropometric measurements were also assessed. *Results*. The IG significantly increased its baseline calcium intake, knowledge and self-efficacy (*P* < 0.001) at post-assessments. A significant decrease in waist circumference in the IG was noted (*P* < 0.05) after intervention. PAL significantly increased by 12.3% at post-test and by 29.6% at follow-up among intervention adults when compared to the CG (*P* < 0.001). *Conclusion*. A theory-driven educational intervention is effective in improving the dietary calcium intake, knowledge, self-efficacy, and PAL of older community-based Mauritian adults.

## 1. Introduction

Low calcium intake is prevalent among older persons due to several factors, namely, decreased mechanical loading of the skeleton, a decline in physical activity, decreased skeletal mass, reduced efficiency of calcium food utilization, and a decline, itself, in food intake [1]. Importantly, a compromised calcium status predisposes one to a range of chronic diseases, including various types of cancer, hypertension, cardiovascular diseases, infectious diseases, metabolic disorders, and inflammatory and autoimmune disorders as well [2]. Moreover, since the skeleton serves as the nutrient reserve for calcium, any decline in calcium intake means a reduced bone mass and ultimately decreased bone strength [1].

In particular, osteoporotic fractures are known to affect a majority of older adults [3]. With a rise in life expectancy of the elderly population worldwide [4] and in Mauritius as well [5], concerns have been raised about the potential increases in bone fracture rates [3] and its associated medical costs such as the need for long-term care and excess mortality [6]. In the United States alone, it is projected that by 2020, medical expenditures related to osteoporosis will be $25.3 billion annually [6]. Therefore, it is imperative to develop a nutrition education (NE) program against calcium deficiency risks so as to curb their associated health care costs and to safeguard older adults' quality of life.

Calcium intake and physical activity are the two most prominent modifiable risk factors associated with osteoporosis [7]. So far, in Mauritius, there is a dearth of interventions aimed at promoting the consumption of calcium-rich foods among older adults. Low calcium intakes reported in several target populations, namely, adolescents [8], low socioeconomic status individuals [9], and, particularly, among older post-menopausal women, are of concern [10]. Over the last two decades, a shift in the dietary habits and lifestyle of Mauritians along with rapid industrialisation has also resulted in an increase in obesity [11]. Additionally, only 16.5% of Mauritian adults, aged 25–74 years, undertook adequate physical activity according to the non-Communicable Disease Survey 2009 [12]. Studies, mentioned herein, highlight the urgency of a calcium and osteoporosis NE program within the Mauritian population.

Calcium NE interventions have been reported to improve quality of life, treatment adherence, and increasing calcium intake among older adults with diagnosed or undiagnosed osteoporosis [13]. Calcium and osteoporosis educational interventions are also highly cost-effective as they result in cost savings through fewer fracture-related events, lower rates of bone loss, maximisation of the positive effect of physical activity on bone health, improvement of muscle performance, and reduced risk of falls among older adults [14]. Emphasis should also be laid upon on increasing calcium intake among high-risk older populations, that is, those having below 400 mg/d, rather than increasing the calcium intake of those already consuming higher amounts [15].

The most fundamental issue in educational interventions has been cited as the selection of the most appropriate theory or model which the NE program will be based upon [16]. The theoretical framework that provided the backbone of the current study was based upon the health belief model (HBM). The forte of HBM, in comparison to other models, lies in its efficiency in preventing diseases through NE programs which promote specific actions (example, increase intake of calcium) and which help participants to take action against the prevention of an unhealthy condition, for example, osteoporosis [17]. HBM-driven NE programs have been demonstrated to significantly increase calcium intake and physical activity in a number of studies [7, 13, 16, 18, 19]. However, none of these studies has targeted an older Mauritian population.

The influence of sociodemographic factors on calcium intake among older adults was also studied. In Mauritius, there has not been any study targeting specifically the relationships which exist between sociodemographic characteristics and calcium intake. Knowledge of socioeconomic and demographic influences on calcium intake is crucial, especially in the development and implementation of practical calcium NE programs and public nutrition policies [20]. For instance, NE and policy making should not be directed at the entire older Mauritian population when, in fact, sub-groups of older adults may have varying calcium intakes [20]. Moreover, sociodemographic characteristics that place certain groups of older adults at the greatest risk for poor calcium diet quality can be identified, so that the risk factor profiles of these at-risk subpopulations can be improved through NE programs [21]. Hence, sociodemographic determinants of calcium intake are worth consideration so as to gain insights into designing successful nutrition education strategies.

The current study aims at improving consumption of calcium-rich foods among older adults through a nutrition education program and at gaining a better understanding of how sociodemographic factors predict calcium intake in this target group. The main objectives of this study are as follows:

1. to evaluate the effectiveness of an educational program in improving dietary calcium intake and physical activity level,
2. to assess any difference in knowledge, health beliefs, and self-efficacy pertaining to calcium intake prior to and after the educational program among older adults,
3. to determine the effects of sociodemographic factors on calcium intake of the participants.

## 2. Materials and Methods

### 2.1. Study Design

This study used a pre-test and a posttest experimental model, with random assignment of one community centre to the intervention group (IG), that is, those who received the educational materials plus the nutrition education (NE) lessons and another community centre to the control group (CG), that is, those who got the educational materials only [17, 19, 22]. The CG (*n* = 91) completed pre-, post-, and follow-up assessments at the same time as the IG (*n* = 98).

### 2.2. Participants

Social welfare or community centres in urban regions of Mauritius were contacted on their official numbers [23]. Two community centres were randomly selected from those which agreed to participate in the NE program. Names of the community centres were kept anonymous as per the request of the participants. Samples of 189 (*n*) women and men, aged 40 years and older, were recruited through voluntary participation in the two community centres [3]. The value of each sample size in each women or men group ranged from 44 to 52. A power analysis (*α* = 0.05; power = 0.80) suggested that a minimum of 84 participants was to be included in each intervention or control group so as to detect a significant 10-point increase in calcium frequency scores of the participants. The formula was derived from Bland [24]. Variance and mean calcium frequency scores used in the calculation were based upon a pilot-test intervention program conducted among 15 adults for 2 weeks.

*Inclusion Criteria.* (i) Participants were men or women aged 40 years or above; (ii) adults who were not mentally disable. Exclusion criteria are (i) having a known diagnosis of osteoporosis or having a chronic disease such as renal failure, heart disease, or diabetes mellitus; (ii) participants having a serious hearing and eyesight problem; and (iii) those who had previously attended a calcium or osteoporosis-related NE program.

### 2.3. Outline of the Calcium Nutrition Education Program

The NE program was conducted from November 2012 till June 2013. Participants in the intervention group were informed to take part in weekly sessions, each of them lasting for two hours on average. The number of participants for each session was 98. A 2-hour duration of a session was considered reasonable as previously done by Liu et al. [25] and Chan et al. [18]. Each session consisted of the use of posters and/or pamphlets distributions and session topics were adapted from Huang et al. [3] and Contento [26]. Comprehension of the educational materials was pretested in a sample of 15 older adults prior to the NE program. Details of the sessions are as follows.

*Session 1*. The objectives of the NE program were introduced and discussed with the participants. Voluntary participants also came forward and shared their knowledge, perceptions, and apprehensions regarding low calcium intake and risk of osteoporosis and chronic diseases. The health belief model and its different constructs were briefly outlined. 

*Session 2*. Calcium poster and pamphlet were utilised to disseminate the benefits of increasing calcium intake and the daily amount needed by postmenopausal women and older adults aged above 40 years. Each individual in the intervention group received his or her own copy of the poster and pamphlet. Individual NE exercise: the intervention group was also asked to write down everything they ate and drank in the past 24 hours. Then, they circled each food or beverage high in calcium and crossed out those low in calcium. 

*Session 3*. Use of the osteoporosis poster and pamphlet to promote awareness on osteoporosis and its link to calcium deficiency, aging, and menopause, preventive strategies such as benefits of exercise and adequate calcium intake to bone health were discussed. The importance of fruits and vegetables was also highlighted since they contained equally protective nutrients like potassium, magnesium, vitamins A, K, E, and C, and carotene in the prevention of chronic diseases and for bone health. Likewise, simple osteoporosis prevention exercise, developed by Hans et al. [27], was demonstrated to maximise retention of calcium in the bone. Simple exercises like daily brisk walking for ten to fifteen minutes and climbing the stairs at home were also covered.

*Session 4*. Barriers to calcium intake such as the cost, preference, cholesterol or fat content, ability to change dietary habits, and digestive response to calcium intake were addressed. 

*Session 5*. Ideas on how to reduce unhealthy snacks and fast food items and substitute them with healthier options like milkshakes, cheese or green leafy vegetables salad with salad and sandwiches, frozen yogurt, milk in soups and whole grain snacks were brainstormed. Given the rise in non-communicable diseases in Mauritius, emphasis was also laid upon the selection of low fat dairy products, for example, low fat/low salt cheese and skimmed milk. A short group (3 or 4 members) presentation of ten minutes was assigned to the participants with the main theme “My Daily Calcium.” 

*Session 6*. A summary of the NE program was communicated. The group presentation was then conducted. After the presentation, the individual NE exercise, previously carried out in Session 2, was repeated with one additional task of writing at least two specific actions which they will take during the following days to increase their own calcium intake.

All talks during the six sessions were addressed in the local language (Creole), so that they could be easily understood and put into practice by all participants.

### 2.4. Questionnaire

A 35-item coded self-administered questionnaire, which was previously pilot-tested, was given to participants. The questionnaire served as pretest and posttest assessment along with a follow-up assessment of the calcium intake, osteoporosis health beliefs, osteoporosis self-efficacy, and knowledge before and after the program, respectively. The calcium food frequency questionnaire (FFQ), adapted from Barr [28] and Hung et al. [29], was included to assess the diet quality of participants concerning calcium-rich foods with the following modifications: (i) soft drinks, pizza, and muffin were excluded from the FFQ since the FFQ aimed to assess frequency consumption of calcium-rich foods only and (ii) “paneer” (Indian cottage cheese), tofu, soy milk and fruits such as grapes and papaya were included as they also contain a significant amount of calcium according to the USDA [30] and are easily available in Mauritius. This section consisted of one more question on the number of glasses of milk drunk daily, as previously assessed by Heaney [31], who stressed upon the importance of evaluating improvements in milk consumption after an education program. A score was assigned to the frequencies of consumption in the FFQ so as to get a good comparison of scores for the total food items before and after the NE program. For example, a score of “4” was assigned to “more than once daily” while a score of “0” was assigned to “rarely/never.” The total possible scores ranged from 0 to 94. Questions on smoking and alcohol consumption were included in this section since they are known risk factors for calcium deficiency and osteoporosis [32, 33] along with calcium supplementation assessed [34]. Weekly physical activity level was also assessed [35] because exercise is linked to calcium intake and bone health [32]. The osteoporosis health belief scale (OHBS), developed by Lee and Lai cited [36], was utilised to obtain information pertaining to participants' susceptibility, severity, benefits to exercise, benefits to calcium intake, barriers to exercise, barriers to calcium intake, and health motivation. The OHBS was shortened to produce a 20-item Likert scale to ease answering. A 5-point Likert scale was used to rate each item. Agree or strongly agree are correct answers [32]. Questions were adapted from Savin [37] to assess participants' knowledge on calcium intake and exercise related to bone health. A score of 1 was assigned to the correct answer (“True”) while a score of 0 was assigned to inability of the participant to answer the question or for incorrect answers (“False” or “Do not know”). The total scores ranged from 0 to 8.

### 2.5. Sociodemographic Data

Information concerning gender, menopausal status, age, ethnicity, occupational status, level of education, personal monthly income, monthly household or family income, and anthropometric measurements was gathered. Menopausal status female participants were asked to select from three options, describing their menopausal status [38]: (i) premenopausal (monthly menstrual bleeding), (ii) perimenopausal (irregular bleeding during past twelve months), and (iii) postmenopausal (last menstrual bleeding at least one year previously). Age groups of older adults were classified according to the following categories: (i) 40–50, (ii) 51–60, (iii) 61–70, and (iv) >70. Ethnic group categories were categorised into the Indo-Mauritians, the Creoles, the Franco-Mauritians, and the Sino-Mauritians [39]. Occupational status was coded according to the system of the British Office of Population Censuses and Surveys, previously used in Mauritius, by Pereira et al. [40]. The following groups were then derived: (i) professional/managerial, (ii) technical/clerical, (iii) skilled worker/artisan, (iv) partly skilled, (v) unskilled and elementary, and (vi) retired individuals. The sample was stratified by four categories of level of education, namely, primary, secondary, tertiary and postgraduate levels. The monthly income groups were classified as follows [11]: (i) low income (less than Rs. 5 000 (≤$161)), (ii) middle-low income (Rs. 5 000–10 000 ($161–$323)), (iii) middle income (Rs. 10 000–20 000 ($323–$645)), and (iv) high income (above Rs. 20 000 (>$645)). Household income categories were classified as follows: (i) low income (less than Rs. 15 000 (≤$484)), middle-low income (Rs. 15 000–Rs 20 000 ($484–$645)), middle income (Rs. 20 000–Rs 3 000 ($645–$968)), and (iv) high income (above Rs 30 000 (>$968)). Onset of average household income category was taken to be Rs. 20 000 [41]. The upper and lower limits of each category were determined taking into account the ranges used by Appanah et al. [11] for personal income level. Socioeconomic status (SES) of the participants was categorised into low, medium, and high SES groups [10]. A scoring system for SES was set up and applied to each socioeconomic indicator. For example, the lowest personal income level (<Rs 5 000) was given a score of 1 and the highest personal income level (above Rs 30 000) was given a score of 5. The scores for each socioeconomic indicator were then added to get a total score for each SES category as follows: (i) low SES (score 4–8), (ii) mediumSES (score 9–12), and (iii) high SES (13–16).

### 2.6. Anthropometry

All measurements were taken using the same instruments to reduce error. Height was measured to the nearest 0.05 m (standard error ± 0.05 m) and weight was measured to the nearest 0.5 kg (standard error ± 0.5 kg) in light clothing and without shoes [42]. Waist and hip girths were measured to the nearest 0.1 cm [43]. BMI was calculated to one decimal place and classified into underweight (<18.5 kg/m^2^), healthy weight (18.5–24.9 kg/m^2^), overweight (25.0–29.9 kg/m^2^), and obese (≥30.0 kg/m^2^) using the WHO [44].

### 2.7. Participantpost Evaluation Questionnaire

A series of questions were adapted from [18, 45] to assess the intervention group participants' satisfaction, motivation, and problems faced regarding the education program. They were also asked to report any other preferences they had for delivery methods of a NE program and to write their suggestions for improving the design of the NE program.

### 2.8. Statistical Analysis

Cronbach's alpha was used to determine the reliability of the scales used in the questionnaire, with a minimum of 0.70 considered appropriate [46]. Cronbach's alpha coefficients for the OHBS and the self-efficacy scale were 0.86 and 0.69, respectively. The Statistical Package for Social Scientist, SPSS software version 16.0 for Windows, was used to conduct statistical analyses at an alpha level of 0.05 and graphs were generated using Microsoft Office Excel 2007. Demographic data were explained using descriptive statistics. A Mann-Whitney test was used to examine differences in (i) calcium intake and anthropometric measurements between intervention and control adults at baseline and at post-test and during follow-up (end-points of the NE program) and (ii) differences in calcium frequency scores for gender. A Wilcoxon signed rank test was used to determine changes in calcium intake, health beliefs, self-efficacy, and knowledge from baseline to post-test and from baseline to follow-up in the intervention and control groups. A Chi-squared test of independence was used to explore the relationships between (i) end-points of the NE program and variables like milk consumption, smoking, alcohol intake, calcium supplement intake, and PAL; and (ii) sociodemographic factors and milk consumption. A Kruskal-Wallis test was used to determine differences in calcium intake among sociodemographic categories. A multiple linear regression model was used to (i) determine the effect of the HBM constructs (perceived susceptibility, perceived seriousness, perceived benefits, perceived barriers, and self-efficacy) and knowledge on calcium frequency scores and (ii) assess the effect of sociodemographic factors on calcium intake.

## 3. Results

### 3.1. Participant Characteristics

Table 1 shows the mean anthropometric measurements for both male and female participants in the study (*n* = 189). Notably, mean anthropometric values for men were greater than those for women. For instance, mean waist circumference (WC) and weight of men were 87.0 (6.72) cm and 70.9 (11.2) kg, respectively while those of women were 85.1 (11.3) cm and 63.3 (13.3) kg. 32.3% of the 189 participants were overweight and 12.2% of them were obese. 9.5% of them were underweight and 46.0% were of normal weight. A final sample of 189 participants (men: 47.6%; women: 52.4%) completed the set of questionnaires at all the three assessment periods (baseline, end-of-program, and follow-up). The majority of participants were Indo-Mauritians (76.7%) with women mostly being post-menopausal ones in both the intervention group (*n* = 25) and control group (CG) (*n* = 17). The majority of adults in the intervention group (IG) were aged from 51 to 60 (19.0%), compared to 18.0% in the control group (CG) who were aged from 40 to 50. For most of the participants, the highest level of education completed was secondary level (61.9%) and 29.6% of them were retired individuals. The most common income level, for both groups, was Rs 5 000–Rs 10 000 ($161–$323).

### 3.2. Intervention Group versus Control Group

#### 3.2.1. Anthropometric Measurements

The intervention group (IG) and the control group (CG) did not differ significantly in terms of weight and body mass index (BMI), *P* > 0.05. Mean hip circumference of IG was significantly lower than that of the control subjects at baseline and post intervention (*P* = 0.001). At posttest, waist circumference (84.6 (9.99)) cm of intervention adults was significantly slightly lower than that of the CG (87.2 (8.49)) cm, *P* = 0.04.

#### 3.2.2. Comparison of Calcium Consumption, Health Beliefs, Self-Efficacy, and Knowledge

At baseline, the CG scored significantly higher than the intervention one on frequency of calcium consumption, perceived benefits, and self-efficacy, (*P* < 0.05) while the intervention adults demonstrated significantly higher perceived susceptibility scores than the control, (*P* = 0.001). After intervention, the IG participants showed statistically significant higher mean calcium frequency scores, perceived susceptibility, self-efficacy, knowledge, and lower perceived barriers scores than the control (*P* ≤ 0.001). During follow-up, IG adults scored additionally higher on perceived benefits (*P* = 0.006) but not at posttest. No significant differences for mean perceived seriousness scores were noted between intervention and control participants at baseline, posttest, and at follow-up, respectively (*P* > 0.05).

### 3.3. Effects of Education Program on Calcium Intake, Health Beliefs, Self-Efficacy, and Knowledge

Table 2 presents the changes in calcium intake in the intervention and control groups over time. From baseline to post-test, IG adults increased their calcium frequency consumption and this increase was sustained during follow-up too, *P* < 0.001. The CG also increased their calcium intake from baseline to posttest (*P* = 0.001), with no increase in calcium intake retained at follow-up (*P* > 0.05). All scores for the different constructs of the calcium and health beliefs scale along with knowledge increased significantly for the IG from baseline to posttest and from baseline to follow-up (Table 2), *P* < 0.001. Mean perceived barriers score decreased from baseline to follow-up. Mean perceived susceptibility scores and mean knowledge scores of the CG also increased significantly from baseline to follow-up (Table 2), *P* < 0.05. Daily consumption (≥2 times) of full cream milk and cheese increased. Consumption of yogurt, grapes, legumes, and almonds once per daily also increased from baseline to follow-up. Weekly consumption of full cream milk, low fat milk, cheese, frozen dairy products, soy milk, orange, grapes, broccoli, bok choy, green vegetables, and fish with bones also increased from baseline to follow-up. All variables were transformed into normal data. A multiple regression was run to predict calcium intake from the different constructs of the health belief model and knowledge. These variables significantly predicted 16.0% of the variability in calcium intake among older participants in the IG posttest, *P* < 0.001 and *R* ^2^ = 0.160. Only perceived seriousness and perceived barriers had significant independent effects on calcium intake (*P* < 0.01). No significant associations were obtained for the CG in this model predicting calcium intake. After controlling other variables, an increase in perceived seriousness scores led to an increase in calcium intake while an increase in perceived barriers scores was associated with a decrease in calcium intake. From baseline to posttest, the percentage of IG participants who drank milk 2 times daily increased by 24.4% compared to 4.4% in the CG. From posttest to follow-up, a slight increase of 1.1% in milk consumption was noted in the IG. At every stage of the nutrition education (NE) program, a significantly moderate association was established between participants and consumption of milk daily whereby a higher percentage of intervention adults consumed milk twice daily (*P* < 0.001).

### 3.4. Changes in Osteoporosis Risk Factors, Supplement Intake, and Physical Activity Level (PAL) over Time

As shown in Table 3, the percentage of smokers remained almost the same in both the groups. The percentage of smokers in the IG remained significantly higher than that in the CG (*P* = 0.005). Alcohol consumption in both groups also did not change throughout the NE program though the percentage of those who consumed alcohol weekly was significantly higher among CG respondents (*P* = 0.003). Alcohol consumption was significantly associated with SES (*P* = 0.015), with 25.3% of low SES older adults consuming alcohol weekly compared to 14.1% of medium SES and 14.0% of high SES participants. Intake of calcium supplements was higher among control respondents at any stage of the NE program (*P* = 0.044) (Table 3). At baseline, a greater number of control participants (69.2%) practiced any kind of physical activity than intervention ones (52.0%). From posttest to follow-up, the percentage of intervention adults who practiced physical activity increased from 64.3% to 81.6%. From baseline to posttest and from baseline to follow-up, daily PAL (1/2 to 1 hour) in the IG increased significantly, *P* < 0.001.

### 3.5. Sociodemographic Factors, Calcium Frequency Scores and Milk Consumption

As shown in Table 4, significant differences in mean calcium frequency scores (*P* < 0.01) were observed for gender, menstrual status, age, occupation status, educational level, personal income level and socioeconomic status (SES). Mean calcium frequency scores of men (31.0 (6.40)) were higher than those for women (28.8 (5.77)), *P* = 0.007. Adults aged ≥ 71 reported a higher mean calcium frequency score (33.4 (7.06)) than the other groups (*P* < 0.001). Postgraduate adults had the highest calcium frequency scores (35.0 (2.69)), *P* = 0.001, while participants of medium SES (31.0 (5.99)) scored higher than high SES ones (26.0 (6.16)), *P* < 0.001. Except for age and ethnicity, all sociodemographic factors were significantly associated with daily milk consumption (*P* < 0.05). 36.4% of women and 37.0% of adults aged ≥ 71 consumed 2 glasses of milk daily (*P* < 0.05). Daily consumption of milk was the highest among the professional group (41.7%), postgraduate adults (55.6%), upper-middle personal income level (54.1%), and lower-middle household income participants (45.6%), *P* ≤ 0.001. Daily milk consumption was the lowest among peri-menopausal older women (22.2%) and among adults of high SES (18.6%), *P* < 0.001.

Dummy variables were used for each sociodemographic factor in the multiple regression model (Table 5). All the variables significantly predicted 35.3% of the variability in calcium intake of older adults in the present study (*R* ^2^ = 0.353; *P* < 0.001). Independently of all other sociodemographic factors, subjects aged 61–70 followed by being female, predicted lower intakes of calcium (*P* ≤ 0.001). Subjects aged 51–60 also had significantly lower intakes of calcium (*P* < 0.05). Unskilled workers had higher intakes of calcium (*P* < 0.05) when all the factors were controlled (Table 5).

#### 3.5.1. Comparison of Calcium Frequency Scores by Socioeconomic Indicators between Intervention and Control Groups

Significant differences in mean calcium frequency scores were noted for all socioeconomic indicators (*P* < 0.05), except for household income level in both groups at baseline and posttest. No significant differences in mean calcium frequency scores were found for SES among intervention adults and for educational level in the CG (*P* > 0.05). Individuals in the managerial or professional group and adults, who studied till the postgraduate level, scored the highest at post-test and during follow-up (*P* < 0.01). Among control adults, mean calcium frequency scores of skilled workers, low-middle income adults, and individuals of medium SES remained the highest at baseline and at post-test and during follow-up (*P* < 0.01). At follow-up, upper-middle household income adults' mean calcium frequency scores were the highest (*P* < 0.01).

### 3.6. Perceptions and Attitudes of Older Adults in the Intervention Group towards the Nutrition Education Program

Table 6 shows the statements and questions pertaining to the participants' views, degree of satisfaction, methods of presentation, level of motivation, barriers to the implementation of dietary and lifestyle guidelines, and suggestions on improving the NE program, which were asked to the intervention participants (*n* = 98) at the end of the NE program. Most participants found the quality of the NE program to be good (68.4%) while 3.1% of older adults rated the program quality poor. 72.4% were satisfied with the program. The majority of adults liked the use of handouts (87.8%), face to face talks (72.4%), exercise workouts (81.6%) and group presentation (71.4%). 4.1% disliked the handouts and the demonstration of exercise work-outs while only 1.0% disliked the group presentation. 77.6% of adults were motivated to implement the NE guidelines. Most adults (83.7%) reported having no problems in implementing the guidelines. Among barriers which have hindered the participants in implementation of the guidelines were lack of self-motivation (3.1%), dependence on other family members for provision of meals (4.1%), and discouragement from spouse, friends, or family members (4.1%). 2 participants reported that they would have preferred instructional videos throughout the NE program with the rest having no comments. Also, 5.1% participants would have liked the addition of free health check-ups and cooking demonstrations (3.1%) to the NE program.

## 4. Discussion

Low calcium intake among older adults in Mauritius is of concern. Prevalence of obesity is also increasing among older individuals as demonstrated by a significant percentage of overweight (32.3%) and obesity (12.2%) in the present study. However, nutrition education (NE) programs, helping older individuals to increase their consumption of calcium-rich foods and to make informed choices about their diet quality and lifestyle, have received scant attention. Findings from the current study provide evidence that the implementation of a theory-driven calcium NE program, among older Mauritians, is a promising intervention to increase calcium intake and promote physical activity in this target population.

### 4.1. Intervention Group versus Control Group

Results from the present study show that compared to the control group (CG), older adults from the intervention group (IG) had higher calcium consumption frequency at posttest and at follow-up. However, no statistically significant difference in perceived seriousness scores between the intervention and control adults was noted (Table 2). These findings are similar to those reported in other studies which are dealing with calcium and osteoporosis educational programs [22, 47]. Higher calcium intake in the IG can be largely explained by the significantly higher increase in perceived susceptibility scores, self-efficacy scores, knowledge scores, and decrease in perceived barriers than those in the CG at post-test and during follow-up. In particular, higher perceived susceptibility to low calcium intake and risk of osteoporosis, with an increase in self-efficacy, improves calcium intake behaviour [48].

As for anthropometric measurements, no statistical differences in body mass index (BMI) and weight were found though there was a 0.3% increase in mean BMI in the CG after intervention. Additionally, from baseline to post-test, an increase of 0.3% in the overweight category of the control group was observed and from post-test to follow-up, there was an increase of 0.2% of obese adults in the control group. Hip circumference of control adults remained significantly higher than that of experimental participants from baseline to post-test. These findings corroborate those conducted on older women whereby a sufficiently high intake of calcium causes no significant change in body weight [49]. In contrast to the current study, calcium intake was significantly and inversely associated with BMI among Brazilian adults [50]. It is also likely that a change in body weight and BMI, which can be attributed to calcium intake, can only occur over years as reported in a prospective study, conducted over 10 years [51]. Moreover, BMI may not stand as an appropriate indicator of an improvement in the fitness level and body composition profile of current participants in the IG as it does not distinguish well between body fat and muscle mass [52]. Nonetheless, a significant difference in waist circumference (WC) was noted between experimental and control participants after intervention. Similar findings have been postulated by Pimentel et al. [53] who concluded that nutrition counselling may have modest effects on BMI, but more impact on abdominal fat loss, reflected by a decrease in WC. Since WC is closely related to intra-abdominal fat mass, a significant decrease in WC, as reported in this study, potentially reflects changes in obesity risk factors, even in the absence of significant weight loss or BMI reduction [54].

### 4.2. Effects of Education Program on Calcium Intake, Health Beliefs, Self-Efficacy, and Knowledge

Calcium consumption frequency scores of intervention adults increased significantly at posttest and at follow-up (Table 2). Significant increases in perceived susceptibility scores, perceived seriousness scores, perceived benefits, and barriers scores together with an increase in self-efficacy scores were observed as well in the IG (Table 2). These results are in line with other studies employing a theory-driven educational program [16, 18, 22, 47]. Higher calcium frequency scores in the IG can be attributed to the increase in daily consumption (once per daily) of yogurt, grapes, legumes, and almonds and the increase in weekly consumption (≥1 weekly) of full cream milk, low fat milk, cheese, frozen dairy products, soy milk, orange, grapes, broccoli, bok choy, green vegetables, and fish with bones from baseline to follow-up. Similar findings have been reported in older Americans among whom daily intakes of calcium-rich foods, fruits, vegetables, fibre, and fluids increased significantly after intervention [55]. This highlights an important aspect of the NE program that simultaneous intakes of other important nutrient-packed foods are increased along with calcium-rich foods, hence enhancing the overall diet quality of the older adults to a large extent. Increases in the perceptions regarding seriousness, severity, and benefits to calcium deficiency and its related diseases like increased falls and osteoporosis have been reported to be the driving factors in increasing intake of calcium-rich foods [18]. Improvement in nutritional knowledge is another important factor associated with increased consumption of calcium-rich foods among older adults [56]. Results of the multiple regression model demonstrated that all components of the health belief model together with knowledge significantly predicted explained 16.0% of the variability in calcium intake at posttest. In particular, an increase in perceived seriousness resulted in an increase in calcium intake after controlling for other variables. Increased perceived seriousness or severity of calcium deficiency and its related diseases such as osteoporosis was the most important factor in increasing compliance of preventive behaviours among older Mauritians. A decrease in perceived barriers, independently, contributed to an increase in calcium frequency scores. Findings reported herein were consistent with other studies which determined that perceived barriers were the most common factor affecting behaviour [32].

Another interesting finding was that from baseline to follow-up, adults in the CG significantly improved on their perceived susceptibility and knowledge too and an increase in calcium intake was noted in the CG from baseline to posttest. Daily milk consumption among control adults increased as well by 4.4% from baseline to posttest. These results bear similarities to those reported among Chinese adults in the control group [57]. One plausible explanation for this finding could be that the use of educational print materials such as pamphlets is a low cost, appealing, and effective way to improve knowledge and educating older participants in interventions since pamphlets and posters were distributed to control participants at baseline [58]. However, the increase in calcium intake among CG adults was significantly lower than that of the intervention ones at post-test and during follow-up and contrarily to intervention participants, control adults did not retain their increase in calcium intake at follow-up (Table 2). This could be attributed to components of the NE program such as interactive lessons, face to face talks, and group presentations which are effective in promoting sustained calcium intake. At baseline, daily milk consumption in the IG was significantly higher than that in the control one and this trend was retained till post-test and follow-up. Increased milk consumption indicates the successful attempt of the education program in meeting the recommended 2 or more servings of dairy products among older adults [59]. However, calcium supplement use did not alter in the IG with calcium supplementation remaining significantly higher in the control one (Table 3). This can be attributed to the fact that the education program's aim was to increase intake of calcium-rich foods instead of supplements, which is the safest method to improve calcium status of older adults [47]. Moreover, calcium supplementation is not sustainable in the long run in developing countries as compared to the promotion of local calcium-rich foods [60].

### 4.3. Effects of Nutrition Education Program on Lifestyle Factors and Physical Activity Level

0.3% decrease in smoking among intervention adults was reported (Table 3). Interestingly, at baseline, adults in the IG showed less healthy habits such as increased smoking frequency and alcohol intake which could be attributed to distinct health habits among different urban sub-groups. The little success regarding prevention of smoking in this study may be accounted by two factors; namely, (i) in comparison to other successful interventions, this study did not prescribe the use of pharmaceutical aids in addition to counselling [61] and (ii) success rates of smoking cessation are more prevalent among those who have tried to quit smoking previously [62] unlike current participants who participated for the first time in an educational program. Weekly alcohol consumption among control adults remained significantly higher than that among the intervention ones. It is equally important to address the fact that alcohol consumption was quite low in this study and was not necessarily viewed as risk factor to osteoporosis. However, significant underreporting may have occurred in both groups as many older people are ashamed to disclose information regarding their drinking and due to minimal social contacts or networks, drinking problems are more difficult to identify in this cohort [63]. In contrast to other brief interventions, among older adults, which have shown modest effects on short-term reduction of alcohol consumption [64], minimal changes in the IG was noted. Ineffectiveness of addressing alcohol consumption in the present study can be explained by the following: (i) stable drinking patterns achieved at older age are more difficult to change [65]; (ii) weekly alcohol consumption was significantly higher among low SES older adults in this study. It has been reported that middle and high SES adults exert a better control on alcohol consumption and among whom, alcohol interventions can be more successful [66] and (iii) use of personalised reports, booklet on ageing and drinking, and drinking diaries, not employed in this study, have previously proved to be effective in reducing the amount of alcohol consumption in older individuals [67]. At baseline, a higher percentage of control adults practiced any form of physical activity though from baseline to follow-up, the percentage of adults in the intervention group increased from 52.0% to 81.6%. Also, daily minimum PAL increased from 9.5% to 19% from baseline to posttest in the IG. Increased PAL at post-test corroborates results of a heath belief model-based program in Iran [68] and in Taiwan [3]. Increase in PAL in the IG has been ascribed to increased perceived benefits, significant improvements in knowledge level and self-efficacy along with increased social support [3]. However, among older Chinese men in Hong Kong, those in the IG did not report more confidence in doing regular PAL and taking adequate calcium than did the CG although intervention adults were found to be significantly more knowledgeable on these issues after intervention [19]. Besides self-efficacy, personal, social, and financial factors also affect PAL of participants and it has been postulated that since a majority of men do not perceive themselves to be at risk of calcium-deficiency diseases like osteoporosis, they do not engage themselves into osteoporosis preventive behaviours [19].

### 4.4. Sociodemographic Factors, Calcium Frequency Scores, and Milk Consumption

Intake of calcium-rich foods was significantly associated with gender (Table 4). Older men consumed calcium-rich foods more frequently than women. Results from the multiple regression model (Table 5) provided further evidence to demonstrate significantly lower intakes of calcium among female participants when all other sociodemographic factors were controlled for. Corresponding findings demonstrate that 80% to 95% of recommended dietary allowance for calcium is more often met by men than women [69]. This can be explained by the higher consumption of calcium-rich foods like dairy products, nuts, and grains among men [70]. Additionally, a higher level of health-consciousness might have accounted for the highest mean calcium frequency scores observed among males. Even so, daily milk consumption was higher among women. In the current study, daily milk consumption was the highest among pre-menopausal women, followed by post-menopausal and perimenopausal women respectively. A previous study, conducted among post-menopausal women, reported higher consumption of full-cream dairy products among older women due to a greater awareness for osteoporosis prevention [10]. Differences in findings might have occurred as a result of different age groups of women in the two studies since the majority of women in the present study are older (aged ≥ 40 years) than those in the previous study (aged 18–60 years). Although nonsignificant, mean calcium frequency scores were higher among Franco-Mauritians, followed by Afro-Mauritians than among Indo-Mauritians and Sino-Mauritians, respectively. Daily milk consumption was the lowest among Sino-Mauritians and the highest among Afro-Mauritians. A high intake of calcium among Afro-Mauritians may be due to a high frequency of milk consumption in this ethnic group. Interestingly, previous studies have reported a low calcium intake among ethnic populations of African origin due to lactose intolerance, culturally determined food preferences, and established dietary practices [71]. Lack of milk intake among Sino-Mauritians reflects the retention of traditional Chinese diets which consist of calcium sources such as green vegetables, soy bean products, cereals, seeds, and nuts [72].

Occupational status, educational level, and personal income level were significantly associated with calcium intake (Table 4). In particular, intake of calcium-rich foods and milk was low among retired individuals compared to other occupational groups. Further evidence was provided by the multiple regression model whereby age of 61–70 was the most significant predictor of low calcium intake in this study population. In addition, 41.0% of adults aged 61 to 70 were retired individuals. This finding may be justified by a constraining food budget due to retirement [73]. In addition, food calcium intake tends to decline with increasing age [1]. Intake of calcium and daily milk consumption was also the highest among postgraduate individuals, adding to previous findings which reported a significant association between higher educational levels and high density calcium in the diet [74]. The same trend was observed among both intervention and control participants. Moreover, educational level was found to be the only socioeconomic indicator significantly linked to diet quality among middle-aged Mauritian women [75]. Another noteworthy result was that in contrast to other studies conducted in Mauritius [10, 75], higher mean calcium frequency scores and daily milk intake were more prevalent in medium socioeconomic status (SES) and low SES adults. Calcium intake remained the highest in medium SES category of both IG and CG too. Furthermore, while in the IG, lower-middle income adults scored the highest on calcium frequency scores, in the CG, the highest intake of calcium was reported among the low-income ones. A better diet quality among low SES populations in some European countries has been linked to a high consumption rate of domestically produced foods [76]. Hence, relatively lower costs of locally produced calcium-rich foods may have contributed to a higher intake of calcium among low-income Mauritians. Inconsistencies can also be attributed to the use of single socioeconomic indicators [75] with educational level most often employed to represent SES in studies [76].

### 4.5. Perceptions and Attitudes of Older Adults in the Intervention Group towards the Nutrition Education Program

From the answers and comments of the participants after intervention, it is apparent that most of the participants found the NE program to be of a good quality with a majority of them satisfied with the outcomes (Table 6). 77.6% were motivated to implement the guidelines provided to them. Components of the NE program such as demonstration of simple exercise workouts, group presentation, and face to face talks were also appreciated by most participants. Good levels of motivation and satisfaction also reflect the increase in self-efficacy among intervention adults at post-test and during follow-up. These results imply that the NE program effectively led to positive behavioural changes in calcium intake and physical activity among older adults in Mauritius. However, some of the participants faced problems such as dependence on family members to provide them with calcium-rich foods and discouragement from spouse or family members and friends (4.1%). Findings reported herein are also informative to suggest that some of the older adults have difficulty in shopping calcium-rich foods and preparing their own meals. 2% of the participants reported financial constraints as barriers to implementing the NE guidelines as limited income or pension have a negative impact on food budgets [73]. These results demonstrate the need of addressing the familial and financial milieu of older people, besides individuals in NE programs to reap more positive outcomes out of interventions. This is of particular importance since studies found that diet quality is greatly affected by emotional support, family networks, social interaction, and social space among older individuals [77].

Major strengths of the present study were (i) the inclusion of a follow-up assessment of calcium intake among the participants 2 months after the intervention, contrary to other similar studies [7, 17, 22]; (ii) a greater focus on increasing the consumption of local calcium-rich foods which is the most effective way of increasing calcium intake [47], rather than on the intake of calcium supplements, and (iii) use of a theory- based (health belief model) nutrition education program. Three limitations of the current study are as follows: (i) due to time constraints, the calcium nutrition education program was conducted over eight months and hence no significant changes in anthropometric measurements for the intervention group, except for waist circumference, could be found; (ii) though the objectives of this study were to assess multiple outcome measures, no correction for multiple testing, example Bonferroni correction, was done [78] as it can increase the risk of false negatives [79]; and (iii) intake of calcium-fortified foods was not assessed in this study because the number of calcium-fortified foods continues to rise in the market and often and fortification levels vary greatly within a food category [7].

## 5. Conclusions

Participants, who attended the calcium and osteoporosis educational intervention, substantially increased their calcium intake, daily milk intake, and positive health beliefs (increase in perceived susceptibility and self-efficacy and a decrease in perceived barriers) and reported higher knowledge scores than those of the control group after intervention. In addition, physical activity in the intervention group also increased significantly from baseline to post-test. Retention of the increase in calcium intake in the intervention group at 2-month follow-up is also notable. Age group of 61–70, followed by being female, were significant predictors of lower intakes of calcium in the current study.

The current study was the first calcium educational intervention carried out among older adults in Mauritius. This study adds to existing research that a nutrition education program, based upon the health belief model, is practical within the older Mauritian population to promote calcium intake. In addition, this study provides baseline evidence to show that an educational intervention may confer modest beneficial effects to abdominal obesity risk. Moreover, healthy lifestyle education programs for older Mauritians should be tailored to address high-risk behaviours such as alcohol consumption and smoking in this target population. Findings of this study also indicate that calcium nutrition education programs should target specific subgroups of older populations such as retired adults, aged 61 to 70, and women who are socioeconomically deprived. Additional studies should examine the effect of a health belief model-based intervention in a more diverse and larger sample of older Mauritians, so that health professionals can consider the use of theory-driven approaches in their intervention programs.

## Figures and Tables

**Table 1:** Mean anthropometric measurements' values for all participants.

Variables	Mean (*SD)	
	Male (*n* = 90)	Female (*n* = 99)
**WC (cm)	87.0 (6.72)	85.1 (11.3)
***HC (cm)	98.9 (9.68)	98.2 (10.3)
Weight (kg)	70.9 (11.2)	63.3 (13.3)
****BMI (kgm^−2^)	24.7 (3.31)	24.5 (6.15)

*SD: standard deviation; **WC: waist circumference; ***HC: hip circumference; ****BMI: body mass index.

**Table 2:** Changes in calcium frequency scores, health belief model constructs, and knowledge scores over time among intervention and control older Mauritian adults.

Variables	*Int.	**Con.	Time effect (*P*value) (i) Baseline to posttest (ii) Baseline to follow-up	
	Mean (SD)		Int.	Con.
*Calcium frequency scores *				
Baseline	29.4 (5.16)	30.6 (7.22)	**<0.001**	**0.001**
Posttest	33.2 (3.88)	29.8 (6.52)	**<0.001**	0.120
Follow-up	34.2 (3.54)	30.2 (6.69)		

*Health belief model constructs *				
Perceived susceptibility				
Baseline	5.37 (1.57)	4.72 (1.42)	**<0.001**	**<0.001**
Posttest	5.83 (1.54)	4.95 (1.14)	**<0.001**	**<0.001**
Follow-up	5.96 (1.40)	4.97 (1.14)		
Perceived seriousness				
Baseline	13.1 (2.12)	13.4 (2.85)	**<0.001**	1.00
Posttest	13.9 (1.95)	13.4 (3.06)	**<0.001**	0.746
Follow-up	14.1 (2.02)	13.5 (2.66)		
Perceived benefits				
Baseline	30.8 (5.15)	32.9 (4.01)	**<0.001**	0.512
Posttest	33.4 (3.13)	33.0 (3.61)	**<0.001**	**0.010**
Follow-up	34.4 (3.46)	33.2 (3.42)		
Perceived barriers				
Baseline	21.5 (2.84)	19.9 (3.52)	**<0.001**	**0.015**
Posttest	22.1 (2.96)	19.6 (3.64)	**<0.001**	0.664
Follow-up	20.8 (3.26)	19.8 (3.68)		
Self-efficacy				
Baseline	17.9 (2.28)	18.5 (2.18)	**<0.001**	**0.029**
Posttest	19.8 (1.94)	18.4 (2.20)	**<0.001**	0.250
Follow-up	20.0 (1.68)	18.4 (2.40)		

*Knowledge *				
Baseline	4.67 (1.75)	4.98 (1.62)	**<0.001**	**0.005**
Posttest	6.82 (0.82)	4.89 (1.64)	**<0.001**	**0.022**
Follow-up	7.58 (0.66)	5.11 (1.66)		

*Int.: intervention group; **Con.: control group.

Statistical test: Wilcoxon signed rank test.

**Table 3:** Changes in osteoporosis risk factors, supplement intake, and physical activity level (PAL) over time in intervention and control groups.

End-points	Int. (%)	Con. (%)	*P* value	Cramer's V
Smoking	Yes	Yes		
Baseline	17.3	4.40	**0.005**	0.206
Posttest	17.2	4.30	**0.005**	0.206
Follow-up	17.0	4.40	**0.005**	0.206
Alcohol consumption	≥Once per week	≥Once per week		
Baseline	13.3	24.2	**0.003**	0.288
Posttest	13.3	24.4	**0.003**	0.288
Follow-up	13.2	24.1	**0.003**	0.288
Physical activity	Yes	Yes		
Baseline	52.0	69.2	**0.016**	0.176
Posttest	64.3	69.1	0.471	0.052
Follow-up	81.6	69.2	**0.047**	0.144
Calcium supplement	Yes	Yes		
Baseline	3.10	4.40	**0.044**	0.207
Posttest	3.10	4.40	**0.044**	0.207
Follow-up	3.10	4.40	**0.044**	0.207

Statistical test: Chi-squared test of independence.

**Table 4:** Differences in baseline mean calcium frequency scores and milk consumption by sociodemographic factors in the whole sample (IG and CG).

Sociodemographic factor	Mean calcium frequency scores (SD)	*P* value	Daily milk (2 glasses) (%)	*P* value Phi Value
Gender				
Male	31.0 (6.40)	**0.007**	24.4	*P* = 0.032 Phi = 0.216
Female	28.8 (5.77)		36.4	

Menstrual status				
Premenopausal	32.8 (5.19)	**<0.001**	44.8	*P* < 0.001 Phi = 0.430
Perimenopausal	28.8 (6.55)		22.2	
Postmenopausal	26.2 (3.92)		39.5	

Age group				
40–50	31.9 (5.03)	**<0.001**	30.5	*P* = 0.068 Phi = 0.291
51–60	28.9 (5.88)		26.6	
61–70	25.8 (4.90)		33.3	
≥71	33.4 (7.06)		37.0	

Ethnicity				
Indo-Mauritian	29.5 (5.91)	>0.05	26.9	*P* = 0.360 Phi = 0.229
Afro-Mauritian	31.1 (7.13)		45.9	
Franco-Mauritian	31.5 (6.75)		27.2	
Sino-Mauritian	28.8 (5.03)		0.0	

Occupational status				
Professional	32.2 (3.54)	**<0.001**	41.7	*P* < 0.001 Phi = 0.653
Technical/clerical	25.3 (4.84)		16.3	
Skilled worker	34.0 (5.40)		41.4	
Partly skilled	28.6 (5.16)		38.2	
Unskilled	32.5 (5.57)		33.3	
Retired	30.8 (6.36)		28.6	

Education level				
Primary	29.7 (4.83)	**0.001**	0.0	*P* = 0.001 Phi = 0.394
Secondary	30.4 (6.50)		32.5	
Tertiary	27.3 (5.30)		35.7	
Postgraduate	35.0 (2.69)		55.6	

Personal income level				
≤Rs 5 000	30.7 (5.72)	**<0.001**	22.9	*P* < 0.001 Phi = 0.611
Rs 5 001–Rs 10 000	32.1 (6.05)		30.6	
Rs 10 001–Rs 20 000	29.4 (4.92)		54.1	
>Rs 20 000	26.0 (6.06)		19.0	

Household income level				
≤Rs 15 000	29.1 (5.18)	>0.05	32.0	*P* < 0.001 Phi = 0.556
Rs 15 001–Rs 20 000	31.0 (4.82)		45.6	
Rs 20 001–Rs 30 000	30.6 (6.63)		22.0	
>Rs 30 000	28.7 (7.04)		22.7	

SES				
Low SES	30.9 (5.65)	**<0.001**	24.0	*P* < 0.001 Phi = 0.542
Medium SES	31.0 (5.99)		45.1	
High SES	26.0 (6.16)		18.6	

Statistical tests: Mann-Whitney test and Kruskal-Wallis test (calcium frequency scores); Chi-square test of independence (milk consumption).

**Table 5:** Sociodemographic correlates of calcium frequency scores from multiple regression in the whole sample.

Sociodemographic variables	*B* (SE)	*P* value
Female	−4.65 (1.35)	0.001
Age groups		
51–60	−3.11 (1.24)	0.013
61–70	−6.89 (1.54)	<0.001
>70	−0.31 (1.57)	0.842
Ethnicity		
Afro-Mauritian	1.09 (1.13)	0.335
Franco-Mauritian	1.78 (2.86)	0.534
Sino-Mauritian	2.50 (3.27)	0.446
Occupation		
Professional/Managerial	3.86 (4.26)	0.366
Technical/Clerical	1.99 (2.77)	0.472
Skilled worker	3.26 (2.20)	0.140
Partly skilled	−0.82 (2.61)	0.754
Unskilled worker	4.35 (1.96)	0.028
Retired	2.46 (1.83)	0.181
Education level		
Primary	0.88 (1.84)	0.633
Tertiary	−3.21 (1.74)	0.067
Post-graduate	0.18 (4.03)	0.964
Personal income level		
Rs 5 001–Rs 10 000	2.24 (1.48)	0.133
Rs 10 001–Rs 20 000	−0.69 (2.22)	0.757
>Rs 20 000	−1.59 (2.53)	0.531
Household income level		
≤Rs 15 000	2.82 (2.70)	0.297
Rs 20 001–Rs 30 000	−0.51 (1.63)	0.756
>Rs 30 000	0.60 (1.83)	0.742

*R* = 0.598; *R* ^2^ = 0.357; *P* < 0.001. Reference variables were excluded to prevent perfect collinearity: gender (male), age group (40–50), ethnicity (Indo-Mauritian), education level (secondary), personal income level (<Rs 5 000), and household income level (Rs 15 000–Rs 20 000).

**Table 6:** Perceptions and attitudes of intervention participants regarding the NE program.

Statements	Frequency	
	*n*	%
Quality of the NE program received		
Excellent	16	16.3
Good	67	68.4
Fair	12	12.2
Poor	3	3.1

Satisfaction concerning the NE program		
Very satisfied	22	22.4
Satisfied	71	72.4
Fairly satisfied	5	5.1
Not satisfied	0	0.0

Use of handouts (posters and pamphlets)		
Excellent	6	6.1
Good	86	87.8
Fair	2	2.0
Poor	4	4.1

Face to face talks		
Excellent	17	17.3
Good	71	72.4
Fair	9	9.2
Poor	1	1.0

Demonstration of simple exercise work-outs		
Excellent	10	10.2
Good	80	81.6
Fair	4	4.1
Poor	4	4.1

Group presentation of which you were part of		
Excellent	18	18.4
Good	70	71.4
Fair	9	9.2
Poor	1	1.0

Level of motivation to implement guidelines		
Very motivated	15	15.3
Motivated	76	77.6
Neutral	5	5.1
Not motivated at all	2	2.0

Problems encountered in guidelines implementation		
Lack of self-motivation	3	3.1
Felt you had to drastically change your diet	2	2.0
Dislike eating calcium-rich foods regularly	1	1.0
Dependent on family member(s) for meal provision	4	4.1
Discouragement from spouse, family, or friends	4	4.1
Financial constraints	2	2.0
No problems	82	83.7

Other preferred ways of NE program delivery		
Short instructional or motivational videos	2	2.0
No other preferences	96	98.0

Suggestions to improve the NE program		
Free bone mass density scan	4	4.1
Free overall health check-ups	5	5.1
Cooking sessions with recipes provided	3	3.1
No suggestion	86	87.8
